# Update: Influenza Activity — United States and Worldwide, May 18–September 20, 2014

**Published:** 2014-10-03

**Authors:** Lenee Blanton, Lynnette Brammer, Sophie Smith, Desiree Mustaquim, Craig Steffens, Anwar Isa Abd Elal, Larisa Gubareva, Henrietta Hall, Teresa Wallis, Julie Villanueva, Xiyan Xu, Joseph Bresee, Nancy Cox, Lyn Finelli

**Affiliations:** 1 Influenza Division, National Center for Immunization and Respiratory Diseases, CDC

During May 18–September 20, 2014,* the United States experienced low levels of seasonal influenza activity overall. Influenza A (H1N1)pdm09 (pH1N1), influenza A (H3N2), and influenza B viruses were detected worldwide and were identified sporadically in the United States. In August, two influenza A (H3N2) variant† viruses (H3N2v) were detected in Ohio. This report summarizes influenza activity in the United States and worldwide during May 18–September 20, 2014.

## United States

The U.S. influenza surveillance system is a collaboration between CDC and federal, state, local, and territorial partners, and uses eight data sources to collect influenza information (1), six of which operate year-round: 1) U.S. World Health Organization (WHO) collaborating laboratories, 2) the National Respiratory and Enteric Virus Surveillance System, 3) reports of human infections with novel influenza A viruses, 4) the U.S. Outpatient Influenza-Like Illness Surveillance Network, 5) the 122 Cities Mortality Reporting System, and 6) the Influenza-Associated Pediatric Mortality Reporting System.§

During May 18–September 20, WHO and National Respiratory and Enteric Virus Surveillance System collaborating laboratories in the United States tested 66,006 specimens for influenza; 3,209 (4.9%) were positive for influenza (Figure). Of the 3,209 specimens positive for influenza during the summer months of 2014, a total of 1,728 (54%) were influenza A viruses, and 1,481 (46%) were influenza B viruses. Influenza B viruses were reported slightly more frequently than influenza A viruses from late May until early July, and influenza A viruses were more commonly reported from mid-July through September. Of the 1,728 influenza A viruses, 1,114 (64%) were subtyped: 45 (4%) were pH1N1 viruses, 1,067 (96%) were influenza A (H3N2) viruses, and two (0.2%) were H3N2v viruses. Influenza viruses were reported from the District of Columbia, Guam, Puerto Rico, and 47 states in all 10 U.S. Department of Health and Human Services regions.¶

During May 18–September 20, data from the U.S. Outpatient Influenza-Like Illness Surveillance Network indicated that the weekly percentage of outpatient visits to health care providers for influenza-like illness (ILI)** remained below the national baseline†† of 2.0%, ranging from 0.8% to 1.4%. The percentage of deaths attributed to pneumonia and influenza (P&I), as reported by the 122 Cities Mortality Reporting System, remained below the epidemic threshold§§ and ranged from 5.2% to 6.0%. Five influenza-associated pediatric deaths occurring during May 18–September 20 were reported; two were associated with an influenza A (H3N2) virus, one was associated with an influenza A virus for which no subtyping was performed, and two were associated with an influenza B virus.

## Novel Influenza A Virus Infection

During May 18–September 20, two human infections with H3N2v viruses were reported by Ohio. Both patients recovered, but one of the two patients was hospitalized as a result of H3N2v illness. In both instances, direct contact with swine in the week preceding illness onset was reported. No ongoing community transmission of these viruses has been detected.

## Worldwide

During May 18–September 20, typical seasonal patterns of influenza activity occurred in temperate climate Southern Hemisphere countries. In Australia and New Zealand, influenza activity began to increase in late July and remained elevated through mid-September. Influenza A viruses predominated in both countries. Although pH1N1 viruses were identified more frequently than influenza A (H3N2) viruses, the proportion of influenza A (H3N2) viruses reported in Australia increased during August to mid-September. Influenza B viruses were reported in much smaller numbers from both countries. In South Africa, influenza activity began to increase in late May and decreased in early August. Influenza A (H3N2) viruses predominated in that country, but pH1N1 and influenza B viruses also were reported. In temperate countries of South America, influenza activity began to increase in June, remained elevated through July and mid-August, and decreased in September. Influenza A viruses were reported more frequently than influenza B viruses, and influenza A (H3N2) viruses were predominant in Chile, Argentina, Uruguay, and Paraguay. In temperate climate countries of Europe and North America, influenza activity was low, and small numbers of pH1N1, influenza A (H3N2), and influenza B viruses were identified.

What is already known on this topic?

CDC collects, compiles, and analyzes data on influenza activity year-round in the United States. The influenza season generally begins in the fall and continues through the winter and spring months; however, the timing and severity of disease and the predominant viral strains can vary by geographic location and season.

What is added by this report?

Worldwide, influenza activity during May 18–September 20, 2014, was elevated in the temperate Southern Hemisphere and tropical regions, compared with their levels outside the usual influenza season. In the United States, low levels of seasonal influenza activity were detected. In August, two influenza A (H3N2) variant viruses were detected; both cases were associated with direct contact with swine.

What are the implications for public health practice?

Annual influenza vaccination is recommended in all persons aged ≥6 months to prevent influenza and its associated complications. Although vaccination is the best way to prevent influenza, treatment with influenza antiviral medications can reduce severe outcomes of influenza, especially when initiated as early as possible, in patients with confirmed or suspected influenza.

In countries with tropical influenza seasonality, overall influenza activity remained low, and the predominant virus type and subtype varied by country. In the Caribbean and Central America, an increase in the number of influenza B viruses was reported in July and August, particularly in Honduras, Jamaica, and Nicaragua, with influenza A viruses cocirculating in Guatemala and Panama. In tropical South America, influenza A viruses were most commonly reported. Influenza A (H3N2) viruses predominated in Brazil and Columbia, whereas influenza B viruses were more frequently reported in Ecuador. In Peru, influenza A (H3N2) and pH1N1 viruses cocirculated, but influenza B viruses also were identified. In South Asia and Southeast Asia, a decrease in influenza activity was observed during August and September, and influenza A (H3N2) predominated in Cambodia, India, China, and Vietnam, with smaller numbers of influenza B viruses reported. In Thailand, influenza B viruses were more frequently reported in July and August, but influenza A (H3N2) and pH1N1 viruses also were identified. During May 1–June 27, 2014, three laboratory-confirmed human cases of influenza A (H5N1) virus infection were reported to WHO; two from Indonesia and one from Egypt (2). During May 1–September 20, 2014, a total of 16 cases of influenza A (H7N9) were identified in China (3).

## Antigenic Characterization of Influenza Virus Isolates

The recommended components for the 2014–15 Northern Hemisphere influenza trivalent vaccines are an A/California/7/2009 (H1N1)-like virus, an A/Texas/50/2012 (H3N2)-like virus, and a B/Massachusetts/2/2012-like (B/Yamagata lineage) virus (4). For quadrivalent vaccines, an additional component, B/Brisbane/60/2008-like (B/Victoria lineage) virus, is recommended (4).

The WHO Collaborating Center for Surveillance, Epidemiology, and Control of Influenza, located at CDC, receives and analyzes influenza virus isolates from laboratories worldwide. CDC antigenically characterized 391 viruses collected during May 18–September 20 from the United States and worldwide, including 70 pH1N1 viruses, 141 influenza A (H3N2) viruses, and 180 influenza B viruses. All 70 (100%) pH1N1 viruses (64 international and six U.S.) were antigenically similar to the A/California/7/2009, the influenza A (H1N1) vaccine component. Of the 141 influenza A (H3N2) viruses characterized (78 international and 63 U.S.), 69 (49%) were antigenically similar to A/Texas/50/2012, the influenza A (H3N2) component of the 2014–15 influenza vaccine for the Northern Hemisphere.

Of the 180 influenza B viruses collected and analyzed during this period (69 international and 111 U.S.), 140 (78%) belonged to the B/Yamagata lineage, and all were antigenically similar to the B/Massachusetts/2/2012 virus, the influenza B component for the 2014–15 Northern Hemisphere trivalent vaccine. The remaining 40 viruses (22%) belonged to the B/Victoria lineage and were antigenically similar to the B/Brisbane/60/2008 virus, the B/Victoria lineage component of the 2014–15 Northern Hemisphere quadrivalent influenza vaccine.

The WHO recommendations for influenza vaccine composition for the 2015 Southern Hemisphere season were made at the WHO Consultation meeting September 22–25, 2014, in Geneva, Switzerland. The recommended components for the 2015 Southern Hemisphere influenza trivalent vaccines are an A/California/7/2009 (H1N1)-like virus, an A/Switzerland/9715293/2013 (H3N2)-like virus, and a B/Phuket/3073/2013-like (B/Yamagata lineage) virus (5). For quadrivalent vaccines, an additional component, B/Brisbane/60/2008-like (B/Victoria lineage) virus, is recommended (5). This represents a change in the influenza A (H3N2) and influenza B/Yamagata lineage components from the 2014 Southern Hemisphere and 2014–15 Northern Hemisphere influenza vaccine formulation.

## Antiviral Resistance Profiles of Influenza Virus Isolates

The WHO Collaborating Center for Surveillance, Epidemiology, and Control of Influenza at CDC tested isolates collected during May 18–September 20 for resistance to influenza antiviral medications. Of the 325 specimens tested for resistance to the neuraminidase inhibitor medications oseltamivir and zanamivir, 111 were collected internationally (16 pH1N1, 61 influenza A [H3N2], and 34 influenza B viruses), and 214 were U.S. specimens (six pH1N1, 99 influenza A [H3N2], and 109 influenza B viruses). None of the tested viruses were found to be resistant to either oseltamivir or zanamivir.

### Discussion

During May 18–September 20, 2014, pH1N1, influenza A (H3N2), and influenza B viruses cocirculated worldwide. It is not possible to predict which influenza virus will predominate or how severe influenza-related disease activity will be during 2014–15 influenza season.

Annual influenza vaccination is the best method for preventing influenza and its potentially severe complications (4). In the United States, an influenza vaccine is recommended for all persons aged ≥6 months without contraindications and can reduce the likelihood of becoming ill with influenza and transmitting the virus to others. Annual influenza vaccination is recommended for optimal protection regardless of whether the vaccine composition has changed since the previous season because immunity wanes over time. For the 2014–15 influenza season, manufacturers have projected a vaccine supply for the U.S. market ranging between 151 million and 159 million doses of vaccine. Although it is difficult to predict the type and subtype of influenza viruses that might circulate during the 2014–15 season, many of the recently examined influenza A (H3N2) viruses show reduced reactivity with sera produced against the A/Texas/50/2012 (H3N2) vaccine virus (the H3N2 component of the 2014–15 influenza vaccine). Vaccination, which includes three or four different influenza viruses depending on the vaccine formulation, is the first line of defense against influenza. Even during seasons when the match between the vaccine viruses and circulating viruses is less than optimal and protection against illness might be reduced, vaccination can offer substantial benefit and might reduce the likelihood of severe outcomes such as hospitalization and death.

Multiple influenza vaccines are approved for use and are being distributed during the 2014–15 season, including a quadrivalent live attenuated influenza vaccine (LAIV4), trivalent and quadrivalent inactivated influenza vaccines (IIV3 and IIV4, respectively), a trivalent cell culture–based inactivated influenza vaccine (ccIIV3), a high-dose trivalent inactivated influenza vaccine (hd IIV3), an intradermally administered IIV3, and a recombinant trivalent influenza vaccine (RIV3). Although both LAIV and inactivated influenza vaccine have been demonstrated to be effective in children and adults, LAIV is approved for use only in persons aged 2 through 49 years with no contraindications or precautions¶¶ (4). In 2014, the Advisory Committee on Immunization Practices recommended the preferential use of LAIV for healthy children aged 2 through 8 years, when it is immediately available, and when the child has no contraindications or precautions (4). However, if LAIV is not immediately available, inactivated influenza vaccine should be used and vaccination should not be delayed to procure LAIV (4). Children aged 6 months through 8 years who are being vaccinated for the first time require 2 doses of influenza vaccine, administered ≥4 weeks apart (6). For children aged 6 months through 8 years who have received influenza vaccination during a previous season, health care providers should consult Advisory Committee on Immunization Practices guidelines to assess whether 1 or 2 doses are required (4).

Although vaccination is the best method for preventing and reducing the impact of influenza, antiviral medications are a valuable adjunct. Treatment with influenza antiviral medications is recommended as early as possible for patients with confirmed or suspected influenza (either seasonal influenza or variant influenza virus infection) who have severe, complicated, or progressive illness; who require hospitalization; or who are at high risk for influenza-related complications*** (7). Antiviral treatment should not be withheld from patients with suspected influenza infection, even if rapid influenza diagnostic test results are negative.

Influenza surveillance reports for the United States are posted online weekly and are available at http://www.cdc.gov/flu/weekly. Additional information regarding influenza viruses, influenza surveillance, influenza vaccines, influenza antiviral medications, and novel influenza A virus infections in humans is available at http://www.cdc.gov/flu.

## Figures and Tables

**FIGURE:**
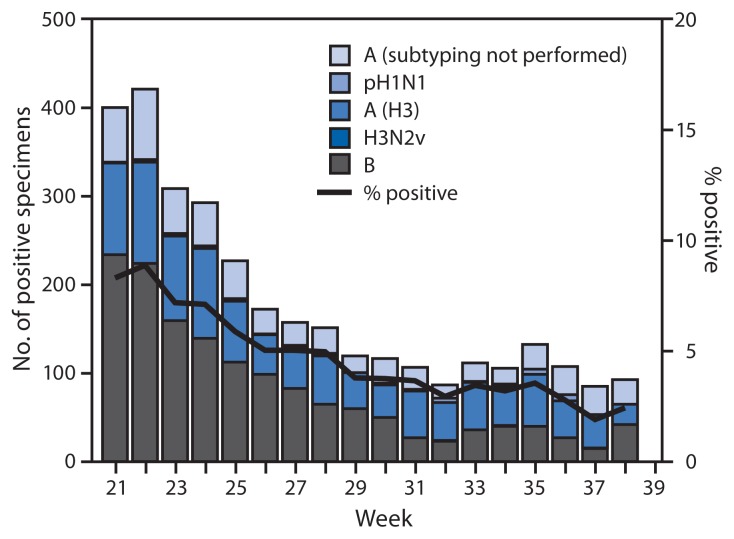
Number* and percentage of respiratory specimens testing positive for influenza reported by World Health Organization and National Respiratory and Enteric Virus Surveillance System collaborating laboratories in the United States, by type, subtype, and week — United States, May 18–September 20, 2014^†^ * N = 3,209. ^†^ As of September 26, 2014.

## Footnotes

* Data as of September 26, 2014.

† Influenza viruses that circulate in swine are called swine influenza viruses when isolated from swine, but are called variant influenza viruses when isolated from humans. Seasonal influenza A (H3N2) viruses that circulate worldwide in the human population have important antigenic and genetic differences from influenza A (H3N2) viruses circulating in swine. Additional information is available at http://www.who.int/influenza/gisrs_laboratory/terminology_ah3n2v.

§ The CDC influenza surveillance system collects five categories of information from the eight data sources: 1) viral surveillance (WHO collaborating laboratories, the National Respiratory and Enteric Virus Surveillance System, and human infections with novel influenza A viruses); 2) outpatient illness surveillance (U.S. Outpatient Influenza-Like Illness Surveillance Network); 3) mortality (122 Cities Mortality Reporting System and influenza-associated pediatric mortality reports); 4) hospitalizations (FluSurv-NET, which includes the Emerging Infections Program and surveillance in three additional states); and 5) summary of the geographic spread of influenza (state and territorial epidemiologist reports).

¶ The 10 regions include the following jurisdictions: Region 1: Connecticut, Maine, Massachusetts, New Hampshire, Rhode Island, and Vermont; Region 2: New Jersey, New York, Puerto Rico, and the U.S. Virgin Islands; Region 3: Delaware, District of Columbia, Maryland, Pennsylvania, Virginia, and West Virginia; Region 4: Alabama, Florida, Georgia, Kentucky, Mississippi, North Carolina, South Carolina, and Tennessee; Region 5: Illinois, Indiana, Michigan, Minnesota, Ohio, and Wisconsin; Region 6: Arkansas, Louisiana, New Mexico, Oklahoma, and Texas; Region 7: Iowa, Kansas, Missouri, and Nebraska; Region 8: Colorado, Montana, North Dakota, South Dakota, Utah, and Wyoming; Region 9: Arizona, California, Hawaii, Nevada, American Samoa, Commonwealth of the Northern Mariana Islands, Federated States of Micronesia, Guam, Marshall Islands, and Republic of Palau; Region 10: Alaska, Idaho, Oregon, and Washington.

** Defined as a temperature of ≥100°F (≥37.8°C), oral or equivalent, and cough and/or sore throat, without a known cause other than influenza.

†† The national baseline is the mean percentage of visits for ILI during noninfluenza weeks for the previous three seasons plus two standard deviations. Noninfluenza weeks are defined as periods of ≥2 consecutive weeks in which each week accounted for <2% of the season’s total number of specimens that tested positive for influenza. The national percentage of patient visits for ILI is weighted on the basis of state population.

§§ The seasonal baseline proportion of P&I deaths is projected using a robust regression procedure in which a periodic regression model is applied to the observed percentage of deaths from P&I that were reported by the 122 Cities Mortality Reporting System during the preceding 5 years. The epidemic threshold is set at 1.645 standard deviations above the seasonal baseline.

¶¶ LAIV should not be used in the following populations: persons aged <2 years or >49 years; those with contraindications listed in the package insert (children aged 2 through 17 years who are receiving aspirin or aspirin-containing products and persons who have experienced severe allergic reactions to the vaccine or any of its components, or to a previous dose of any influenza vaccine); pregnant women; immunosuppressed persons; persons with a history of egg allergy; children aged 2 through 4 years who have asthma or who have had a wheezing episode noted in the medical record within the past 12 months, or for whom parents report that a health care provider stated that the child had wheezing or asthma within the last 12 months; persons who have taken influenza antiviral medications within the previous 48 hours; and persons who care for severely immunosuppressed persons who require a protective environment. Such persons should not receive LAIV, or should avoid contact with immunosuppressed persons for 7 days after receipt, given the theoretical risk for transmission of the live attenuated vaccine virus.

*** Persons at higher risk include 1) children aged <5 years (especially those aged <2 years); 2) adults aged ≥65 years; 3) persons with chronic pulmonary (including asthma), cardiovascular (except hypertension alone), renal, hepatic, hematologic (including sickle cell disease), metabolic disorders (including diabetes mellitus), or neurologic and neurodevelopment conditions (including disorders of the brain, spinal cord, peripheral nerve, and muscle, such as cerebral palsy, epilepsy [seizure disorders], stroke, intellectual disability [mental retardation], moderate to severe developmental delay, muscular dystrophy, or spinal cord injury); 4) persons with immunosuppression, including that caused by medications or by human immunodeficiency virus infection; 5) women who are pregnant or postpartum (within 2 weeks after delivery); 6) persons aged ≤18 years who are receiving long-term aspirin therapy; 7) American Indians/Alaska Natives; 8) persons who are morbidly obese (i.e., with a body mass index ≥40); and 9) residents of nursing homes and other chronic-care facilities.

## References

[1] Brammer L, Blanton L, Epperson S (2011). Surveillance for influenza during the pH1N1 pandemic—United States, April 2009–March 2010. Clin Infect Dis.

[2] World Health Organization (2014). H5N1 highly pathogenic avian influenza: timeline of major events, 14 July 2014.

[3] World Health Organization (2014). Disease outbreak news.

[4] Grohskopf LA, Olsen SJ, Sokolow LZ (2014). Prevention and control of seasonal influenza with vaccines: recommendations of the Advisory Committee on Immunization Practices (ACIP)—United States, 2014–15 influenza season. MMWR.

[5] World Health Organization (2014). Recommended composition of influenza virus vaccines for use in the 2015 southern hemisphere influenza season.

[6] Neuzil KM, Jackson LA, Nelson J (2006). Immunogenicity and reactogenicity of 1 versus 2 doses of trivalent inactivated influenza vaccine in vaccine-naive 5–8-year-old children. J Infect Dis.

[7] CDC (2011). Antiviral agents for the treatment and chemoprophylaxis of influenza—recommendations of the Advisory Committee on Immunization Practices (ACIP). MMWR.

